# Fluorofenidone inhibits apoptosis of renal tubular epithelial cells in rats with renal interstitial fibrosis

**DOI:** 10.1590/1414-431X20198772

**Published:** 2019-10-28

**Authors:** Hui Yang, Weiru Zhang, Tingting Xie, Xuan Wang, Wangbin Ning

**Affiliations:** Department of Rheumatology and Immunology, Xiangya Hospital, Central South University, Changsha, China

**Keywords:** Fluorofenidone, Unilateral ureteral obstruction, Renal fibrosis, Apoptosis, Enalapril

## Abstract

This study aimed to investigate the mechanism of fluorofenidone (AKF-PD) in treating renal interstitial fibrosis in rats with unilateral urinary obstruction (UUO). Thirty-two male Sprague-Dawley rats were randomly divided into sham, UUO, UUO + enalapril, and UUO + AKF-PD groups. All rats, except sham, underwent left urethral obstruction surgery to establish the animal model. Rats were sacrificed 14 days after surgery, and serum was collected for renal function examination. Kidneys were collected to observe pathological changes. Immunohistochemistry was performed to assess collagen I (Col I) protein expression, and terminal deoxynucleotidyl transferase-mediated nick end-labeling staining to observe the apoptosis of renal tubular epithelial cells. The expression of Fas-associated death domain (FADD), apoptotic protease activating factor-1 (Apaf-1), and C/EBP homologous protein (CHOP) proteins was evaluated by immunohistochemistry and western blot analysis. AKF-PD showed no significant effect on renal function in UUO rats. The pathological changes were alleviated significantly after enalapril or AKF-PD treatment, but with no significant differences between the two groups. Col I protein was overexpressed in the UUO group, which was inhibited by both enalapril and AKF-PD. The number of apoptotic renal tubular epithelial cells was much higher in the UUO group, and AKF-PD significantly inhibited epithelial cells apoptosis. The expression of FADD, Apaf-1, and CHOP proteins was significantly upregulated in the UUO group and downregulated by enalapril and AKF-PD. In conclusion, AKF-PD improved renal interstitial fibrosis by inhibiting apoptosis of renal tubular epithelial cells in rats with UUO.

## Introduction

Renal interstitial fibrosis is the common pathway and pathological basis of chronic kidney diseases (CKDs) progressing to end-stage renal disease (ESRD). Its main features include tubular atrophy and deposition of a large amount of extracellular matrix (ECM) (1). The pathogenesis of renal interstitial fibrosis is complex. Studies have found that excessive apoptosis of renal tubular epithelial cells may be the critical cause of tubular atrophy, and persistent apoptosis limits the repair of renal function (2).

Fluorofenidone (1-(3-fluorophenyl)-5-methyl-2-(1*H*)pyridone, AKF-PD) is a novel compound drug designed at Central South University (Changsha, Hunan, China). It has proven to have therapeutic effects in treating multiple-organ fibrosis, such as kidney, liver, and lung (3), but the anti-fibrotic mechanism of AKF-PD is not very clear. Previous studies indicated that AKF-PD could inhibit inflammation (4,5), oxidative stress (6), renal tubular epithelial cell transdifferentiation (7), and fibroblast activation (8). This study aimed to observe the effect of AKF-PD on renal tubular epithelial cell apoptosis and apoptotic signaling pathway in rats with unilateral urinary obstruction (UUO), so as to enhance the understanding of the pharmacological mechanism of AKF-PD.

## Material and Methods

### Animals and model establishment

A total of 32 male Sprague-Dawley rats, aged 6–8 weeks, weighing 180–220 g were purchased from SLAC Laboratory Animal Center (China). All rats were kept in the Experimental Animal Center of Central South University at a temperature of 25±2°C, humidity of 55±2%, and 12-h light/dark cycle. The Animal Center also provided Specific Pathogen Free (SPF) food and drinking water *ad libitum*. This study was approved by the Institutional Animal Care Committee of Xiangya School of Medicine, Central South University.

The rats were randomly divided into sham, UUO, UUO + enalapril, and UUO + AKF-PD groups after 1 week of adaptive feeding, and each group had eight rats. The UUO operation was performed as described in a previous study (9). The rats of the latter three groups underwent left ureteral ligation close to the pelvis under aseptic conditions, while in the sham group, the left ureter was isolated without ligation. The drugs were dissolved in 0.5% carboxymethyl cellulose sodium. Enalapril (10 mg · kg^−1^ · day^−1^; Yangtze River Pharmaceutical Group Co., Ltd., China) and AKF-PD (500 mg · kg^−1^ · day^−1^; Xiangya School of Pharmaceutical Sciences) were given by gavage to rats on the day after surgery. The concentration of AKF-PD was based on our previous studies (8,10). The sham and UUO groups received an equivalent amount of normal vehicle by the same method. All rats were sacrificed 14 days after surgery.

Blood samples were collected by heart punctures in rats under anesthesia. The sera were centrifuged at 956 *g* for 10 min at 4°C. Serum creatinine (Scr) and blood urea nitrogen (BUN) were determined at the clinical laboratory of Xiangya Hospital according to manufacturer’s instructions.

### Histopathological examination

The obstructive kidney tissues were routinely fixed in formalin, embedded in paraffin, and sliced into 4-μm-thick sections. Hematoxylin and eosin (HE) and Masson trichrome staining were used to estimate the degree of renal tubulointerstitial injury and collagen deposition. Ten fields of renal cortex per section were randomly chosen under 200× magnification by a digital camera (Leica, Germany) coupled to a light microscope (Leica DM 5000B, Germany). The images had a high-resolution, and Image-Pro Plus 6.0 software (Media Cybernetics, Inc., USA) was used for semiquantitative analysis. The assessment criteria of renal interstitial injury comprised eight indexes, including renal tubular epithelial cell vacuolar degeneration, tubular dilatation, tubular atrophy, red cell cast, protein cast, interstitial edema, interstitial fibrosis, and interstitial cellular infiltration. The injury index ranged from 0 to 3, with the following definition: 0) normal; 1) mild change; 2) moderate change; and 3) severe change. The renal fibrosis index was evaluated using the scoring system as follows: 0 points: normal; 1 point: <25% staining; 2 points: 25–50% staining; 3 points: 51–75% staining; and 4 points: >75% staining (10).

### Immunohistochemistry

Immunohistochemical staining was conducted for detecting the expression and distribution of Col I, FADD, Apaf-1, and CHOP in the obstructive renal tissue. Shortly after dewaxing and rehydration, the sections were soaked in 3% peroxide at room temperature for 20 min. The gastric enzyme diluent was used for antigen retrieval in an incubator at 37°C, followed by 3% bovine serum albumin to block the sections for 30 min. Then, the sections were incubated with primary antibodies against Col I (1:200; Abcam, UK), FADD (1:200; Abcam), Apaf-1(1:200; Abcam), and CHOP (1:100; Abcam) in phosphate-buffered saline (PBS) overnight at 4°C. The secondary antibody was added for 30 min at 37°C after the sections restored to room temperature naturally. PBS was used as a negative control instead of primary antibodies. Each section was observed under 200× magnification and 10 random fields were preserved. Five sections per group were randomly chosen for this study. The percentage of positive staining area in each field was calculated using Image-Pro Plus 6.0 software. The results were classified as follows: 0 point, normal; 1 point: <25% staining; 2 points: 25–50% staining; 3 points: 51–75% staining; and 4 points: >75% staining (11).

### TUNEL staining

The apoptotic epithelial cells were detected using the TUNEL Reagent Kit (Roche Applied Science, USA). Briefly, paraffin sections were deparaffinized and rehydrated, and blocked in 3% peroxide at room temperature for 30 min. Then, 20 μg/mL proteinase K was used to digest protein for 20 min at 37°C. The sections were incubated with TUNEL reagents in the dark for 60 min at 37°C, followed by transfer into converter-peroxidase in the dark for 30 min. They were washed with PBS three times for 5 min after each step. Then, diaminobenzidine staining and hematoxylin counterstaining were performed. Finally, the sections were sealed using neutral gum. Ten fields were selected randomly from each renal tissue section under 400× magnification, and the number of apoptotic epithelial cells in each field was counted. The average value was calculated as the level of apoptosis. Five slices were chosen from each group.

### Western blot analysis

The renal tissues were lysed in sodium dodecyl sulfate (SDS) lysis buffer (Beyotime, China) for extracting total protein, and the concentration of protein was determined using the bicinchoninic acid protein assay kit (Thermo Fisher, USA). Then, 40 µg total protein was loaded into each lane and separated by SDS-polyacrylamide electrophoresis. The proteins in the SDS gel were transferred onto a polyvinylidene difluoride membrane (Millipore, USA), and the nonspecific binding protein was blocked in Tris-buffered saline with Tween 20 (TBST) buffer containing 5% skimmed milk for 1 h at 37°C. The membrane was incubated using primary antibodies against glyceraldehyde-3-phosphate dehydrogenase (GAPDH, 1:5000; Abcam), FADD (1:100; Abcam), Apaf-1 (1:2000; Abcam), and CHOP (1:1000; Abcam) overnight at 4°C on a shaking table. Sequentially, the membrane was immersed in goat anti-rat immunoglobulin G (IgG; 1:5000; ABclonal, USA) or goat anti-rabbit IgG (1:5000; ABclonal) antibody for 1 h at room temperature. The membrane was washed three times using TBST for 10 min after each step. The protein bands were visualized using an enhanced chemiluminescence system detection kit (ECL; Amersham, USA) in a bioimage system (Bio-Rad, USA). The grayscale value was calculated using Image-Pro Plus 6.0 software. Five samples were selected randomly for western blot analysis.

### Statistical analysis

All data are reported as means±SD, and SPSS 19.0 statistical analysis software (IBM, USA) was used for data analysis. The comparison between groups was performed by one-way analysis of variance. Newman-Keuls multiple comparison test was used as the statistical post-test. A P value <0.05 was considered statistically significant.

## Results

### AKF-PD had no effect on the renal function in UUO rats

**Figure 1.**
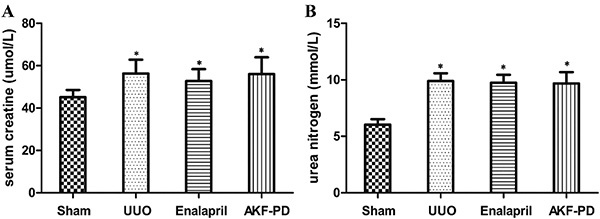
Fluorofenidone (AKF-PD) had no effect on renal function in the unilateral urinary obstruction (UUO) group. **A**, Semiquantitative analysis results of serum creatine and (**B**) urea nitrogen in the sham, UUO, UUO + enalapril, and UUO + AKF-PD groups. Data are reported as means±SD. *P<0.05 *vs* the sham group (ANOVA).

Two rats were excluded from the study due to model failure or ileus-related death during the experimental period. The number of rats in each group at the end of the experiment was seven in the sham and UUO groups and eight in the UUO + enalapril and UUO + AKF-PD groups. Our results showed that Scr and BUN levels were significantly increased in the three experimental groups compared to the sham group (P<0.05). However, no significant difference was observed between the UUO + enalapril group and the UUO + AKF-PD group (P>0.05) (Figure 1A and B).

### AKF-PD alleviated renal pathological injury of obstructed kidney in UUO rats

**Figure 2.**
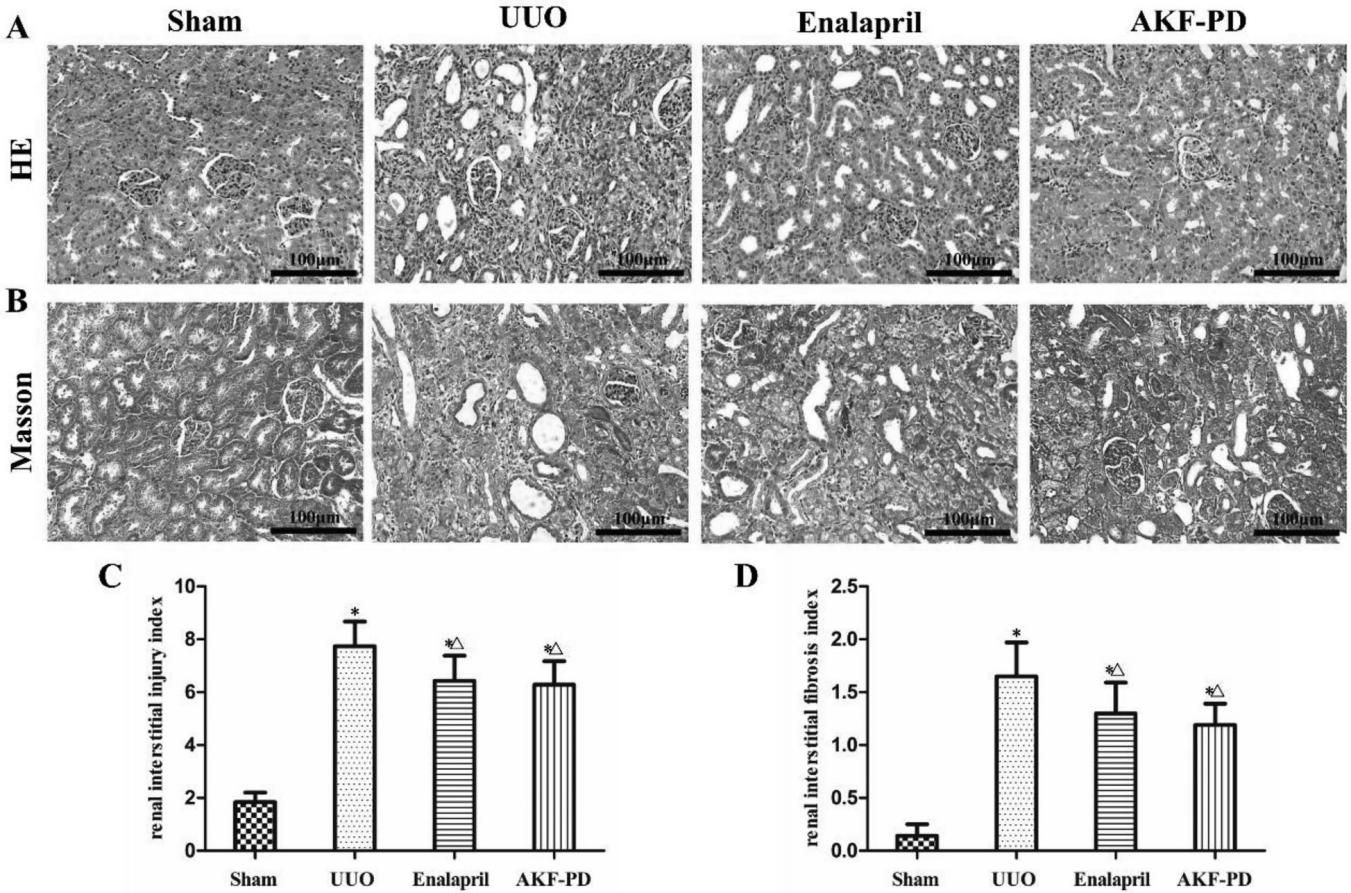
Fluorofenidone (AKF-PD) attenuated renal pathological changes in the unilateral urinary obstruction (UUO) group. **A**, HE staining and **B**, Masson staining results in the sham, UUO, UUO + enalapril, and UUO + AKF-PD groups (200× magnification, bar: 100 μm). **C**, Semiquantitative analysis results of renal interstitial injury index, and **D**, renal interstitial fibrosis index. Data are reported as means±SD. *P<0.05 *vs* the sham group. ^△^P<0.05 *vs* the UUO group (ANOVA).

No significant pathological changes were observed in the sham group under the light microscope. Moreover, vacuolar degeneration of renal tubular epithelial cells, interstitial inflammatory cell infiltration, and a large amount of collagen deposition in interstitial space were also observed in the UUO group. Different degrees of tubular atrophy and dilatation were seen in the kidneys of rats in the UUO group. The lesions were alleviated distinctly after treatment with enalapril or AKF-PD (Figure 2A and B).

The renal interstitial injury index showed that the score of the UUO group increased markedly compared with the sham group (P<0.05) and decreased to various degrees after treatment with enalapril or AKF-PD (P<0.05). Although the index in the UUO + AKF-PD group was slightly lower than that in the UUO + enalapril group, no significant difference was observed between these two groups (P>0.05) (Figure 2C). This study also analyzed the degree of collagen deposition using Masson staining. The renal interstitial fibrosis index was higher in the three experimental groups than in the sham group (P<0.05), and enalapril or AKF-PD significantly reduced collagen deposition to different extents compared with the UUO group (P<0.05). However, the effect in the two treatment groups showed no significant difference (P>0.05) (Figure 2D)

### AKF-PD downregulated the expression of COL I in renal interstitium of UUO rats

**Figure 3.**
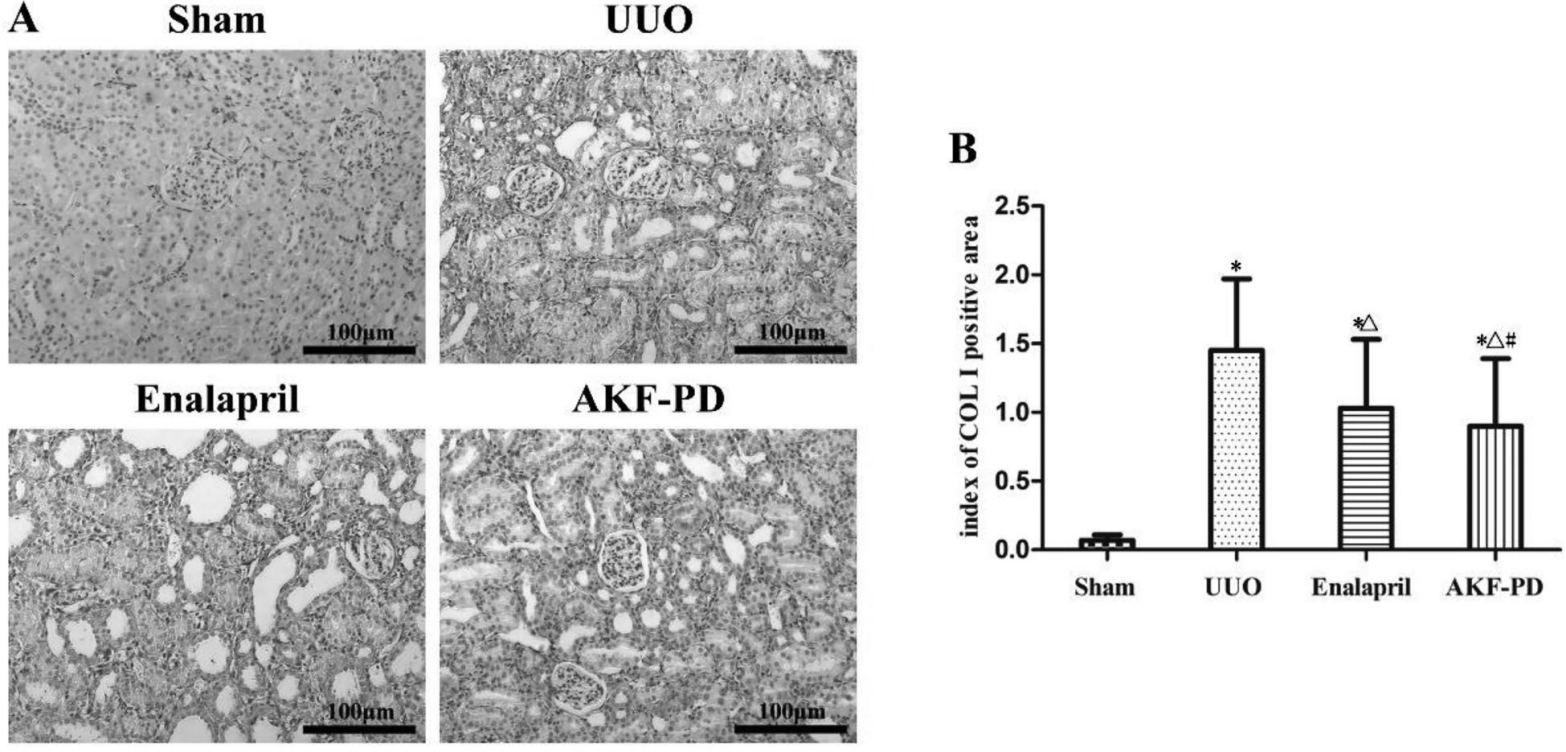
Fluorofenidone (AKF-PD) downregulated the expression of Col I in the renal interstitium of rats in the unilateral urinary obstruction (UUO) group. **A**, Immunohistochemistry results of Col I in the sham, UUO, UUO + enalapril, and UUO + AKF-PD groups (200× magnification, bar: 100 μm). **B**, Semiquantitative analysis results of expression of Col I. Data are reported as means±SD. *P<0.05 *vs* the sham group. ^△^P<0.05 *vs* the UUO group. ^#^P<0.05 *vs* the UUO + enalapril group.

Figure 3A shows that the brown-stained Col I was localized slightly around renal vessels but hardly appeared in the renal interstitial space in the sham group. However, a large amount of Col I was deposited in the renal interstitium of rats in the UUO group. Treatment with enalapril or AKF-PD led to a significant reduction in the interstitial Col I deposition. The semiquantitative results of Col I in immunohistochemical sections are shown in Figure 3B. The expression of Col I was markedly upregulated in the UUO group compared to the sham group (P<0.05). Both enalapril and AKF-PD could significantly downregulate the expression of Col I in the UUO group (P<0.05), but the efficacy of AKF-PD was stronger than that of enalapril (P<0.05).

### AKF-PD reduced the number of apoptotic renal tubular cells in UUO rats

**Figure 4.**
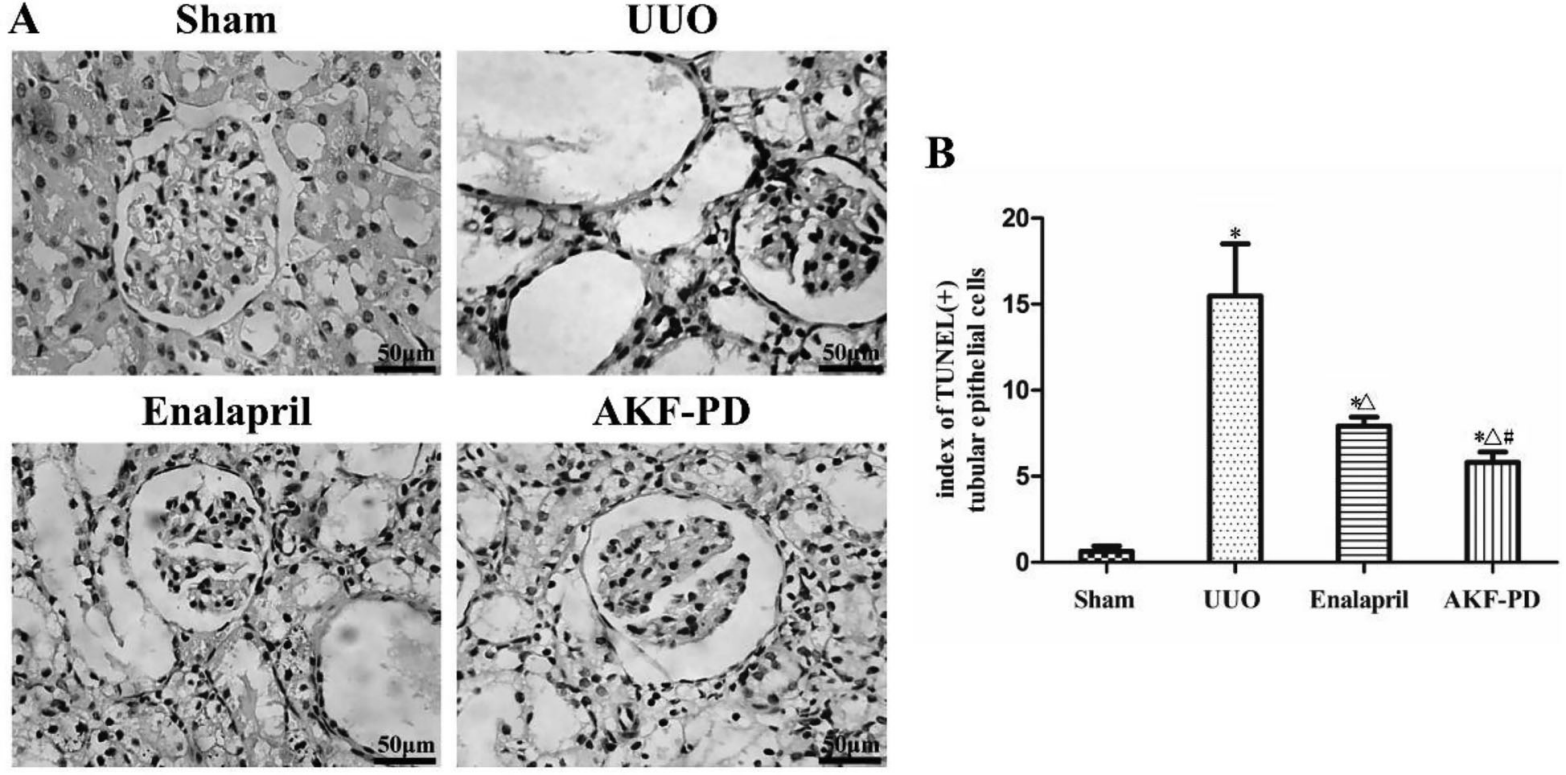
Fluorofenidone (AKF-PD) reduced the number of apoptotic renal tubular epithelial cells in the unilateral urinary obstruction (UUO) group. **A**, TUNEL staining results of kidney tissues in the sham, UUO, UUO + enalapril, and UUO + AKF-PD groups (400× magnification, bar: 50 μm). **B**, Semiquantitative analysis results of the number of apoptotic tubular epithelial cells. Data are reported as means±SD. *P<0.05 *vs* the sham group. ^△^P<0.05 *vs* the UUO group. ^#^P<0.05 *vs* the UUO + enalapril group (ANOVA).

The TUNEL staining sections revealed only a few apoptotic tubular epithelial cells in the sham group, while a large number of positive-stained epithelial cells were seen in the UUO group. These cells mainly existed in the atrophic and dilated tubules. The number of apoptotic cells decreased with the administration of enalapril or AKF-PD, and the therapeutic efficacy of AKF-PD seemed to be significant (Figure 4A). As shown in Figure 4B, the number of apoptotic cells increased in the UUO group compared to the sham group (P<0.05), and enalapril and AKF-PD suppressed tubular apoptosis to varying extents (P<0.05). The reduction was more distinct in the UUO + AKF-PD group than in the UUO + enalapril group (P<0.05).

### AKF-PD decreased the expression of FADD, Apaf-1, and CHOP proteins in kidney tissue of UUO rats

**Figure 5.**
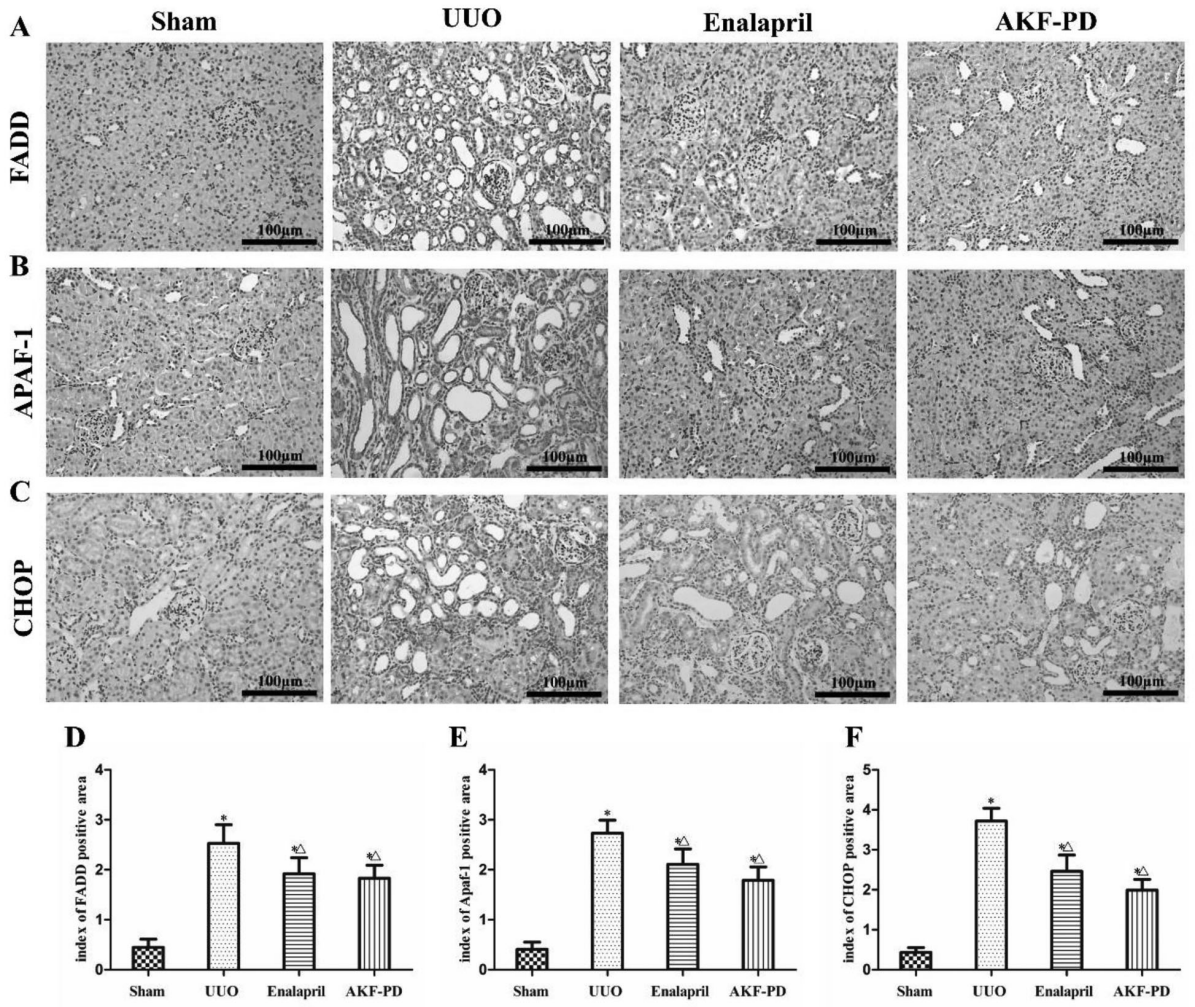
Fluorofenidone (AKF-PD) suppressed the protein expression of FADD, Apaf-1, and CHOP in the renal interstitium of rats in the unilateral urinary obstruction (UUO) group. Immunohistochemical results of FADD (**A**), Apaf-1 (**B**), and CHOP (**C**) in the sham, UUO, UUO + enalapril, and UUO + AKF-PD groups (200× magnification, bar: 100 μm). **D**, **E**, and **F**, Semiquantitative analysis results of the protein expression of FADD, Apaf-1, and CHOP. Data are reported as means±SD. *P<0.05 *vs* the sham group. ^△^P<0.05 *vs* the UUO group (ANOVA).

Immunohistochemical staining showed only low expression of FADD, Apaf-1, and CHOP in the kidney tissue of rats in the sham group (Figure 5A–C). After induction of UUO, the expression of these apoptotic proteins was upregulated significantly in the cytoplasm of atrophic or dilated tubular epithelial cells (P<0.05). Compared with the UUO group, the number of positive-stained cells and protein expression levels of FADD, Apaf-1, and CHOP all decreased in the UUO + enalapril and UUO + AKF-PD group (P<0.05). There was no significant difference between these two treatment groups (P>0.05) (Figure 5D–F).

**Figure 6.**
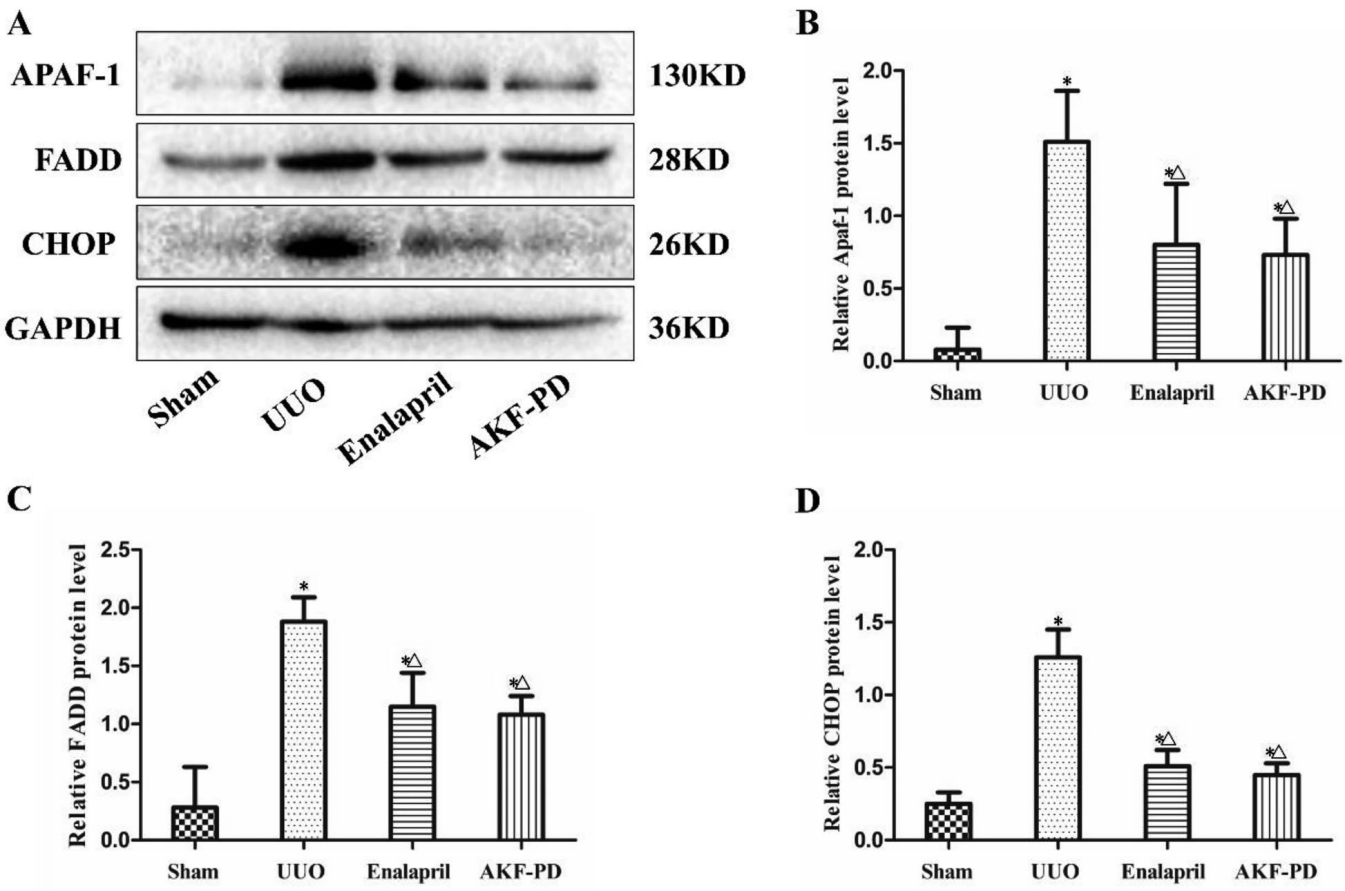
Fluorofenidone (AKF-PD) decreased the protein expression of FADD, Apaf-1, and CHOP in the kidney tissues of the unilateral urinary obstruction (UUO) group. **A**, Western blot analysis results of FADD, Apaf-1, and CHOP in the sham, UUO, UUO + enalapril, and UUO + AKF-PD groups. **B**, **C**, and **D**, Semiquantitative analysis results of the protein expression of Apaf-1, FADD, and CHOP, respectively. Data are reported as means±SD. *P<0.05 *vs* the sham group. ^△^P<0.05 *vs* the UUO group (ANOVA).

To further confirm the effect of AKF-PD on apoptosis-related proteins, the expression of FADD, Apaf-1, and CHOP in the renal tissues of the treatment and control groups was examined by western blot analysis. The developed bands and analysis results are displayed in Figure 6. The aforementioned three proteins were lowly expressed in the sham group, and the expression increased in the rats of the UUO group (P<0.05). After treatment with enalapril or AKF-PD, expression of FADD, Apaf-1, and CHOP distinctly declined in both groups compared with the UUO group (P<0.05), but no statistical difference was found between the two treatment groups (P>0.05).

## Discussion

Renal fibrosis is the common pathway for the progression of various kidney diseases to ESRD, which is irreversible. Hence, inhibiting the process of renal fibrosis is the key to controlling the development of renal failure. Angiotensin-converting enzyme inhibitors and angiotensin receptor blockers are the traditional and widely clinically used renoprotective drugs, but they only delay the progression of renal failure other than blocking or reversing renal fibrosis (12). Patients with already developed ESRD can only be treated by renal replacement therapy, such as dialysis or kidney transplantation. Therefore, a new effective anti-fibrotic medicine with multi-therapeutic pathways and targets is urgently needed.

AKF-PD is a pyridine compound having an anti-fibrotic effect similar to that of pirfenidone (13). Most of the preclinical studies of AKF-PD have been completed, and its application for phase I anti-hepatic fibrosis clinical trials is ongoing. Previous studies showed that AKF-PD had significant effects against renal interstitial fibrosis in many animal models, such as rats with UUO (10) and diabetic nephropathy (14). In this study, we proved that AKF-PD could ameliorate renal interstitial injury and fibrosis. These results indicated that AKF-PD had a renoprotective effect in rats with UUO. Col I is the main component of ECM, and the amount of collagen deposition in the renal interstitium represents the degree of fibrosis. In the present study, the expression of Col I protein was significantly downregulated by AKF-PD, further confirming that AKF-PD could relieve tubulointerstitial fibrosis.

The anti-fibrotic mechanism of AKF-PD is still largely unknown. Previous studies reported that it might occur by inhibiting the transdifferentiation of renal tubular epithelial cells and suppressing the proliferation and activation of fibroblasts *in vivo* (7,8). AKF-PD could also alleviate renal fibrosis by downregulating the expression of fibrogenic cytokines, such as transforming growth factor-β and connective tissue growth factor, and blocking the synthesis of Col I and Col III, promoting the degradation of ECM and inhibiting the oxidative stress and inflammatory response *in vitro* and *in vivo* (3). *In vitro*, AKF-PD could inhibit angiotensin II-induced apoptosis of renal tubular cells via blocking the Fas/FasL pathway (15) and attenuating apoptosis of NRK-52E cells induced by H_2_O_2_ (16).

Previous studies indicated that apoptosis of tubular epithelial cells is important in renal tubulointerstitial fibrosis. It was commonly believed that the main cause leading to tubular atrophy was excessive apoptosis of tubular epithelial cells after injury. The number of apoptotic epithelial cells was found to be significantly higher in patients with CKD than in normal people, and the number of apoptotic cells in renal tissue was related to renal interstitial injury index, renal fibrosis index, level of proteinuria, and deterioration of renal function (17). Other studies also reported that the increase in tubular epithelial cell apoptosis was apparently associated with interstitial fibrosis in UUO-operated rats, whereas inhibiting the apoptosis of tubular epithelial cells improved renal fibrosis (18). In this study, AKF-PD significantly relieved tubular epithelial cell apoptosis in rats with UUO, as detected by TUNEL staining, indicating that AKF-PD might improve renal interstitial fibrosis in rats with UUO by inhibiting the apoptosis of renal tubular epithelial cells.

Three pathways of apoptotic signal transduction, namely death receptor pathway, mitochondrial pathway, and endoplasmic reticulum pathway, have been reported to be involved in the occurrence and development of renal interstitial fibrosis.

The Fas/FasL pathway is the most classical death receptor pathway. During this process, activating FADD is the key step of this signaling pathway (19). The Fas/FasL pathway-related factors, such as Fas, FasL, and FADD, were upregulated in rats with UUO, while blocking the Fas pathway inhibited renal tubular epithelial cell apoptosis and renal interstitial fibrosis (20). Our research revealed that the expression of FADD protein in the renal tissue of rats in the UUO group was significantly reduced after AKF-PD treatment. It suggested that AKF-PD might inhibit renal tubular epithelial cell apoptosis by inhibiting the expression of FADD in the Fas/FasL pathway.

The mitochondrial pathway, also known as the endogenous apoptotic pathway, also participates in the regulation of apoptosis. Apaf-1 activation is an important part of this process (21). Mitochondrial pathway-mediated apoptosis has been demonstrated in many animal models, including rats with UUO, and interrupting this pathway could significantly relieve renal interstitial fibrosis (22). The results of our study showed that the expression of Apaf-1 protein was greatly reduced in renal tissues of rats in the UUO group after AKF-PD treatment. It indicated that AKF-PD might also inhibit renal tubular epithelial cell apoptosis by inhibiting the expression of Apaf-1 in the mitochondrial pathway.

Endoplasmic reticulum pathway, also known as endoplasmic reticulum stress (ERS), has been proven to be involved in the pathogenesis of various fibrotic diseases, including renal fibrosis (23). The ERS-induced apoptosis is highly complex, and it is mainly mediated by CHOP (24). Studies showed that CHOP and other ERS signaling pathway-related molecules are overexpressed in rats in the UUO group, and inhibiting the expression of CHOP could improve renal interstitial fibrosis (25). Our study showed that the expression of CHOP protein was greatly reduced in renal tissues of rats in the UUO group after AKF-PD treatment. This indicated that AKF-PD might inhibit renal tubular epithelial cell apoptosis by inhibiting the expression of CHOP in the endoplasmic reticulum pathway.

Enalapril is a widely used angiotensin-converting enzyme inhibitor in clinics. It has remarkable renoprotective and antifibrotic effects on UUO rats (9). Thus, we chose enalapril as the control in our study. Our previous study showed that AKF-PD, compared to enalapril, had a lower interstitial injury score and a better inhibitory effect on renal fibrosis indices such as Col I protein level, Col III mRNA and protein levels, and TGF-β1 mRNA and CTGF protein expression (10). The present study further demonstrated that AKF-PD significantly decreased Col I protein level compared to enalapril. Also, AKF-PD exhibited a more potent effect of anti-apoptosis in renal tubular epithelial cells compared to enalapril. Moreover, it has been reported that AKF-PD can inhibit renal fibrosis via multiple pathways and targets (3). These results suggest AKF-PD may have advantages over enalapril in improving renal fibrosis.

In summary, AKF-PD inhibited the apoptosis of renal tubular epithelial cells in rats in the UUO group. This effect might be mediated by interfering with the expression of FADD, Apaf-1, and CHOP, which are key molecules in the Fas, mitochondrial, and endoplasmic reticulum pathways, respectively. However, the specific targets of AKF-PD in these three pathways need further investigation. The findings of this study suggested that the inhibition of tubular epithelial cell apoptosis might be a promising strategy for treating renal interstitial fibrosis.
